# State of play in the molecular presentation and recognition of anti-tumor lipid-based analogues

**DOI:** 10.3389/fimmu.2024.1479382

**Published:** 2024-11-28

**Authors:** T. Praveena, Jérôme Le Nours

**Affiliations:** Infection and Immunity Program and Department of Biochemistry and Molecular Biology, Biomedicine Discovery Institute, Monash University, Clayton, VIC, Australia

**Keywords:** CD1d, glycolipids, iNKT cells, α-GalCer, tumor, immunotherapy

## Abstract

The Natural Killer T cells (NKT) are a unique subset of T lymphocytes that recognize lipid-based antigens that are presented by the monomorphic MHC-I-like molecule, CD1d. Over 30 years ago, the discovery of the glycolipid α-Galactosylceramide (α-GalCer) from the marine sponge *Agelas mauritianus*, as a potent activator of the invariant Natural Killer T (iNKT) cells, has attracted great attention for its use in cancer immunotherapy. However, α-GalCer can initiate both pro-inflammatory T helper cell 1 (Th1) and anti-inflammatory Th2 type immune responses that can result in either enhanced or suppressed immunity in a somewhat unpredictable manner. Th1 polarized immune response is often correlated with an optimal anti-tumor immunity, and therefore α-GalCer did not fully offer the desired potential as an anti-tumor therapeutic. Over the past decades, considerable efforts have then been invested into the design and development of novel synthetic α-GalCer analogues that will direct a more efficient immune response towards the production of Th1 biased cytokines. In this minireview, we will discuss how subtle modifications in the chemical nature of a number of α-GalCer derivatives varied immune responses. Whilst some of these analogues showed potential in enhancing stability within CD1d and directing favourable immune responses for tumor immunotherapy, their responses in mice also highlighted the need for further research in humanized models to overcome translational challenges and optimize therapeutic efficacy.

## 1 Introduction

Natural killer T cells (NKT) belong to an unconventional subset of T lymphocytes that recognise a diverse range of lipid-based antigens (Ags) *via* their surface-expressed receptors, the T cell receptors (TCRs) (1). Unlike the presentation of peptidic-derived Ags by the polymorphic Major Histocompatibility Complex I and II (MHC-I and II) molecules (2), these lipid-based Ags are presented by the monomorphic MHC-I-like molecule, CD1d, for NKT TCR recognition and activation (3). There exist two broad classes of NKT cells (type I and II) that differ by their TCR gene usage and Ag specificity (3). Relative to the MHC-I-restricted TCRs, the type I NKT or invariant NKT cells (iNKT) utilize a more limited range of TCR genes, such that human iNKT cells typically express an invariant TRAV10^+^TRAJ18^+^ (or Vα24-Jα18) rearranged TCRα chain and most express a TRBV25-1 (Vβ11) TCRβ chain. An equivalent of type I NKT cells exists in mice whereby the NKT cells express an invariant TCRα chain rearrangement TRAV11^+^TRAJ18^+^ (or Vα14-Jα18), and typically use 1 of 3 different V genes in the TCRβ chain that include TRBV13, TRBV29 and TRBV1 (or Vβ8, Vβ7, Vβ2). When specifically activated *via* their TCR, the iNKT cells produce an array of cytokines that include Th1-, Th2- and Th17-type cytokines, which enables them to influence immune outcomes in a broad range of diseases, and with the greatest interest in their ability to promote tumor immunity (4, 5). This distinctive property of iNKT cells has enabled them to be considered as attractive immunotherapeutic candidates (6).

The signature feature of iNKT cells lies in their ability to recognise the marker glycolipid-based agonist, α-GalCer, first identified and characterized from the marine sponge *Agelas mauritianus* (7). The first synthetic analogue of α-GalCer (KRN7000) was first shown to exhibit NK-mediated anti-tumor effects (8), then shown to be a CD1d-restricted antigen for iNKT cells (9, 10). Over the years, α-GalCer has been widely used experimentally and in preclinical and clinical translational studies (Phase I/II clinical trials) as a potent iNKT cell agonist (11–15). However, a common issue with α-GalCer is that it induces high levels of both proinflammatory (Th1) and anti-inflammatory (Th2) cytokines, which have opposing effects and can reduce its therapeutic efficacy (4). Consequently, there has been significant interest in developing glycolipid analogues of α-GalCer that can skew the immune response in one direction or the other. Some of these analogues are designed to promote Th2-biased responses (IL-4 > IFN-γ) following iNKT cell activation (16, 17), while others favour Th1-biased responses (IFN-γ > IL-4) in the same context (18). Further, α-GalCer elicit a proinflammatory Th1-biased response in mice, and these outcomes did not translate well to systems that model human iNKT cell responses. In addition, it is also known that other populations of ‘atypical’ NKT cell subsets (γδ T cells and δ-αβ T cells) can respond to CD1d-α-GalCer (19–21); Yet, the role of these distinct T cell subsets in affecting tumor immunity remains unknown. Over the past two decades, studies demonstrated the feasibility of using glycolipid analogues to manipulate the iNKT cell response that can translate into more tailored iNKT cell-based therapies (17, 22, 23). Here, we will provide a brief overview on how a range of modified α-GalCer were presented by CD1d and recognised by iNKT TCRs and summarise how those chemical modifications impacted iNKT cell immune responses.

## 2 Structural overview of CD1d/α-GalCer/*i*NKT TCR interactions

**Figure 1:**
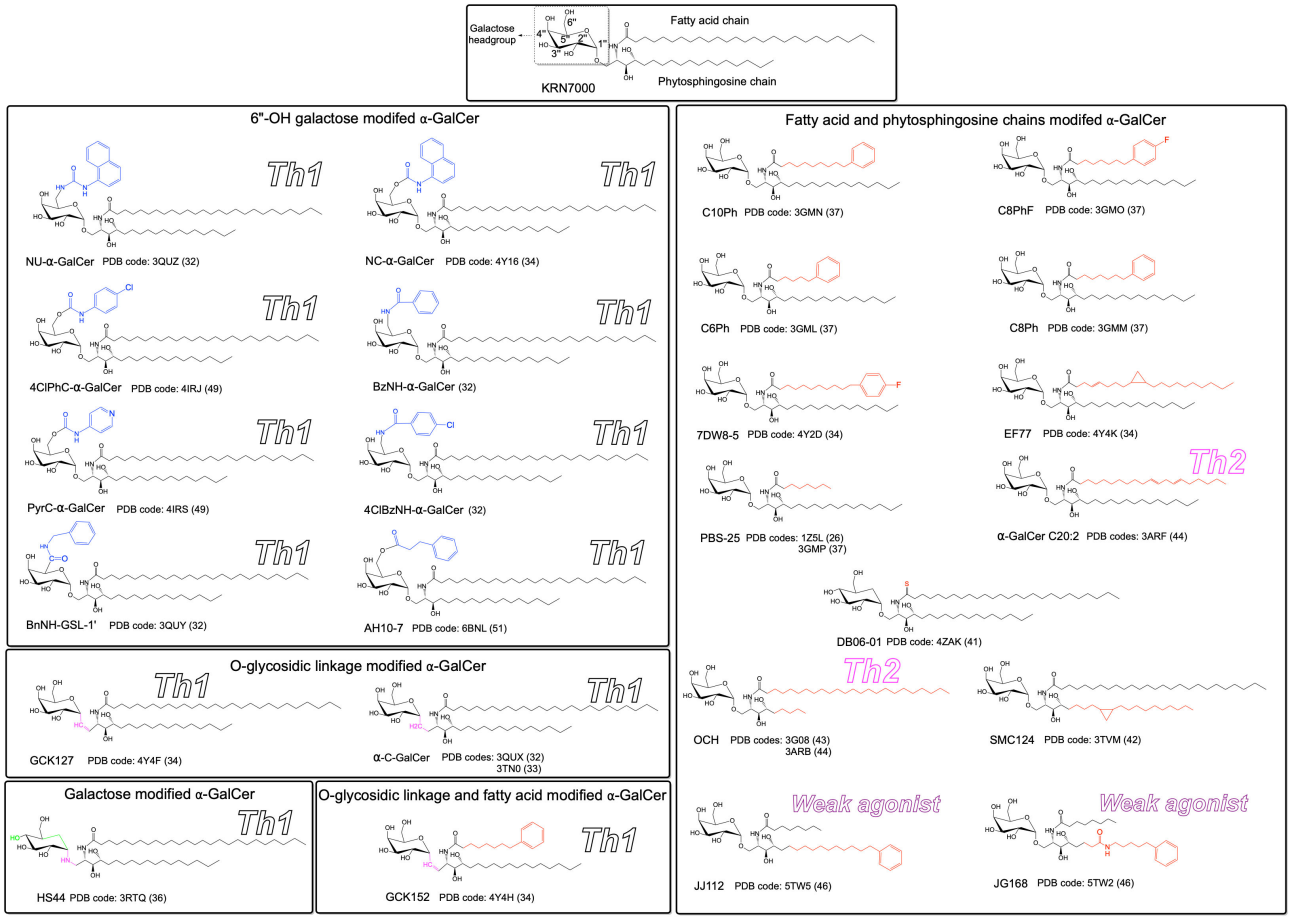
Chemical structures of modified α-GalCer analogues. Relative to the α-GalCer structure, the introduced chemical modifications are colored in blue, red, green and magenta. Analogues were grouped based on the introduced chemical modification. The functional responses (Th1, Th2 or weak agonist) that exhibit clear polarization are indicated for the respective ligand. The PDB code for the known crystal structures are also indicated.

**Figure 2:**
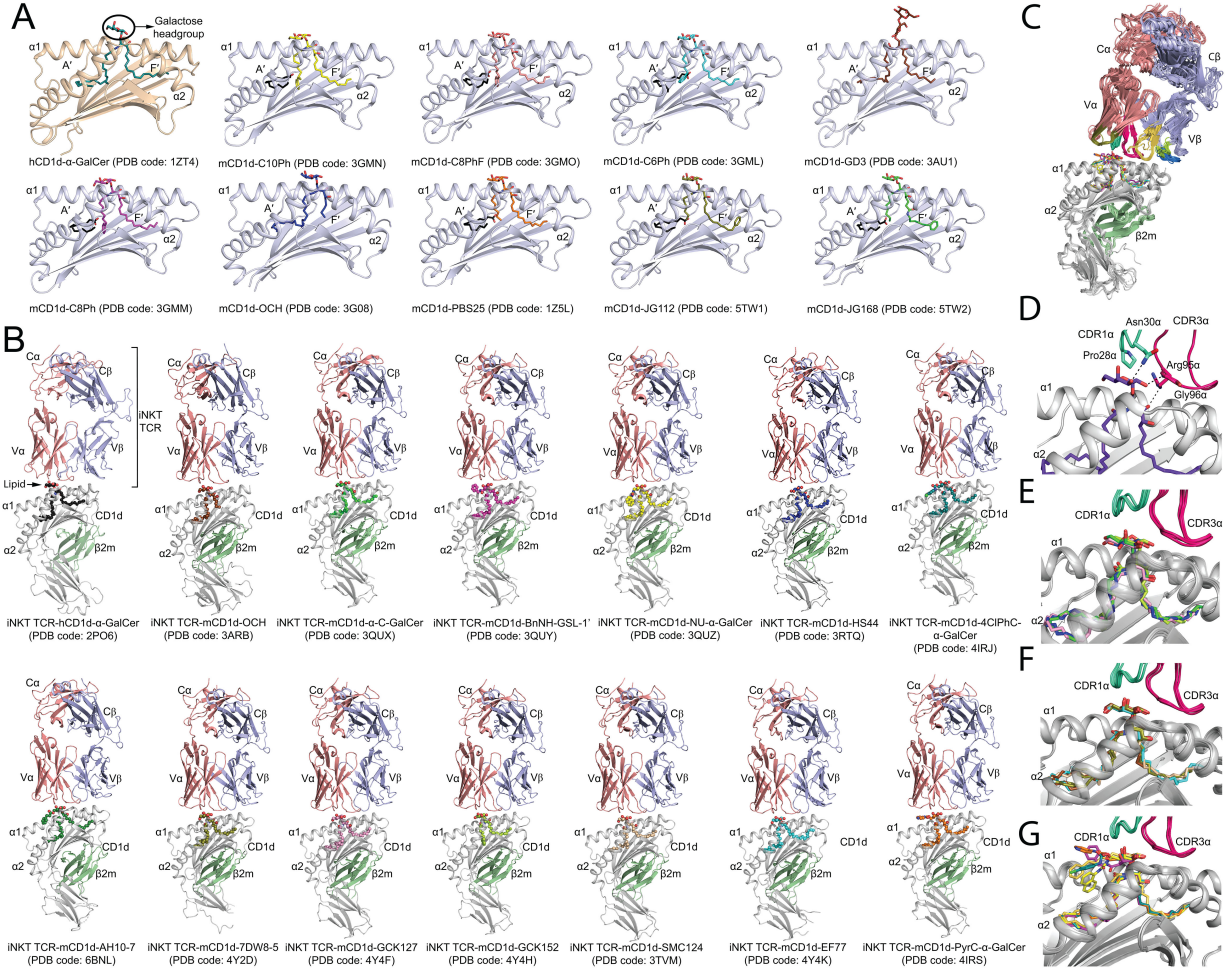
Molecular insights into the presentation and recognition of α-GalCer analogues. **(A)** Cartoon representation of the binding groove of CD1d presenting α-GalCer and modified α-GalCer. The PDB code for each binary complex crystal structure is indicated. The α1/α2 domain forming the hydrophobic binding groove (A′- and F′-pockets) of human CD1d (hCD1d) and mouse CD1d (mCD1d) are shown as cartoon representation in wheat and light blue, respectively. The bound α-GalCer and modified α-GalCer are shown as coloured sticks. Spacer lipids are shown as black sticks. **(B)** Cartoon representation of the crystal structure of iNKT TCR-CD1d-α-GalCer analogues ternary complexes deposited in the protein databank (PDB). CD1d, grey; TCRα, salmon; TCRβ, light blue; β2-microglobulin (β2m), green. The PDB code of the crystal structures are indicated. **(C)** Overall superposition of iNKT TCR-CD1d-α-GalCer analogues ternary crystal structures available in the PDB database. **(D)** Molecular interactions between the iNKT TCR and α-GalCer. Hydrogen bonds are shown as dashed lines. **(E)** Superposition of the bound O-glycosidic linkage modified α-GalCer. **(F)** Superposition of the bound acyl and phytosphingosine chains modified α-GalCer. **(G)** Superposition of the bound 6′′-OH galactose modified α-GalCer. The overall positioning of the CDR1α and CDR3α loops of the iNKT TCR are also shown in **(E–G)**.

Until now, the molecular presentation and/or recognition of a total of over 20 modified α-GalCer analogues have been investigated. Structurally, α-GalCer consists of a α-D-galactose headgroup that is covalently linked to both fatty acid and phytosphingosine chains *via* a O-glycosidic bond (**Figure 1**). The first crystal structure of hCD1d-α-GalCer (24), mCD1d-α-GalCer analogue (25), iNKT TCR-hCD1d-α-GalCer and iNKT TCR-mCD1d-α-GalCer complexes (26, 27) provided detailed insights into the molecular basis that underpinned the presentation of the lipid-based antigen α-GalCer by CD1d and its subsequent recognition by the iNKT TCR (**Figures 2A, B**). In brief, the structures revealed that the fatty acid and phytosphingosine chains of α-GalCer sit deep within the hydrophobic A′- and F′-pockets of CD1d, respectively, while the polar galactose moiety protrudes out of the CD1d binding cleft for recognition by the iNKT TCR (**Figure 2A**). Here, the iNKT TCRs adopted a highly conserved strategy of parallel mode of docking over the F′-pocket of the CD1d binding groove (**Figures 2B, C**) whereby the Complementary Determining Regions (CDR) of the TCRα chain (CDR1α and CDR3α) made key interactions with the 2′′-, 3′′- and 4′′-hydroxyls of α-GalCer and the TCRβ chain mediated interactions with CD1d. Several CD1d residues also interacted closely with the O-glycosidic oxygen, the 2′′- and 3′′-hydroxyls of α-GalCer, thus stabilizing the antigen within the CD1d cleft (**Figure 2D**). These structural insights into the CD1d/α-GalCer/iNKT TCR molecular interactions provided a highly valuable rational basis to design novel classes of α-GalCer analogues that may impact the iNKT TCR recognition and direct a more favourable Th1 skewed immune response for cancer immunotherapy (**Figure 1**). In brief, the introduced chemical modifications have been mainly targeting three distinct parts of the α-GalCer molecule that included the O-glycosidic bond, the glycolipid hydrophobic tails, and the galactose 6′′-hydroxyl (**Figure 1**).

## 3 O-glycosidic modified α-GalCer analogues

The synthetic α-C-GalCer was the first developed α-GalCer analogue in which the O-glycosidic oxygen of α-GalCer was replaced by a carbon to confer resistance to the enzymatic cleavage of the O-glycosidic linkage by α-galactosidases and stabilize the glycolipid (**Figure 1**) (18, 28). Interestingly, in mice, α-C-GalCer exhibited a superior effect against melanoma metastases in the lungs than α-GalCer (18) and also directed the iNKT stimulation towards a strong Th1 biased production (29) although this activation was much weaker in humans. In 2011, the crystal structures of the iNKT TCR-mCD1d-α-C-GalCer ternary complex (30, 31) revealed that the iNKT TCR adopted a docking strategy identical to the one observed in the iNKT TCR-mCD1d-α-GalCer complex (**Figure 2B**). However, the number of molecular interactions of α-C-GalCer with mCD1d and the TCR was reduced relative to α-GalCer. This structural observation was also consistent with the observed loss of affinity and shorter half-life (t_1/2_) of mCD1d-α-C-GalCer for the iNKT TCR. More recently, an *E*-alkene linker was introduced in α-GalCer in place of the glycosidic link to generate the “GCK” series of analogues (GCK127 and GCK152) (**Figure 1**) (32). Similarly to α-C-GalCer, though GCK127 and GCK152 induced a strong IL-2 secretion in the cell-free antigen presentation assay, they failed to robustly activate human cell lines. At the molecular level, GCK127 interactions with mCD1d and the iNKT TCR were highly conserved with hydrogen bonds formed between the galactose and the CDR1residues Asn30α, Arg95α and Gly96α of the TCRα chain (32). On the other hand, GCK152 shows reduced interaction with the TCR due to the loss of hydrogen bonds between 4′′-OH and Asn30α, and the 3′′-OH and Arg95α. In the continuing search for novel and potent α-GalCer analogues, a series of molecules termed aminocyclitols was developed and that included the HS44 ceramide in which an amide linkage and a carbon cyclitol ring replaced the glycosidic bond and the galactose moiety, respectively (**Figure 1**) (33, 34). By comparison to α-GalCer, the affinity of the iNKT TCR for HS44 was significantly weaker and this correlated with a loss of hydrogen bond between the 4′′-OH of HS44 and Asn30α of the CDR1α loop of the iNKT TCR. Whilst subtle structural differences were observed within the TCR-lipid interactions, the overall positioning of the galactose headgroup and the CDRα loops was very similar across all the ternary complexes (**Figure 2E**).

## 4 Fatty acid and phytosphingosine modified α-GalCer analogues

By anchoring the lipid α-GalCer deep within the A′- and F′-pockets of the CD1d binding groove, the hydrophobic fatty acid and phytosphingosine chains play key roles in stabilizing the lipid within the antigen-presenting molecule and in orienting the carbohydrate polar headgroup for recognition by the iNKT TCR. Thus, both aliphatic chains were attractive targets for chemical modifications in order to generate novel synthetic analogues that may direct more efficiently and robustly the stimulatory profile towards a Th1-mediated immune response. The glycosphingolipid PBS25 was the first fatty acid modified α-GalCer analogue to be characterized (**Figures 1**, **2**) (25, 35). Soon after, the structural observation revealing that the contour of the A′-portal of CD1d was delineated by a number of accessible bulky aromatic residues (24, 25) led to the generation of novel synthetic α-GalCer analogues (“CPh” series) including 7DW8-5 (36–38) (**Figure 1**). Here, the α-GalCer fatty acid chain was modulated to shorter lengths and flanked by a terminal aromatic moiety (Phenyl) that was introduced to favour aromatic lipid/CD1d interactions, and thus enhance the overall glycosphingolipid stability within the binding pockets of CD1d (**Figure 1**). Analogues containing a 6 to 10 carbons long fatty acid chain promisingly exhibited a more potent human NKT cell-mediated Th1 cytokine response than α-GalCer. The crystal structure of the mCD1d in complex with a series of CPh analogues (C6Ph, C8Ph, C8PhF and C10Ph) provided molecular insights into the presentation of this new class of glycosphingolipids by mCD1d (35) (**Figure 2**). In brief, the shorter modified fatty acid chains adopted different conformations in the A′-pocket, however, these introduced modifications did not impact the overall positioning of the solvent-exposed galactose moiety and the sphingosine chain in the F′-pocket (**Figure 2F**). Synthetic glycolipids such as EF77, SMC124 and DB06-1 harbouring novel fatty acid or sphingosine chain modifications (32, 39, 40) were also assessed for their ability to stimulate iNKT cells. EF77 and SMC124 were synthetic derivatives of the naturally occurring plakoside A glycolipid that contained a cyclopropyl group on either the acyl chain or sphingoid base (32, 39) whilst the synthetic lipid DB06-01 was similar to α-GalCer in which the C2 carbonyl oxygen on the acyl chain was replaced by a sulphur atom (**Figure 1**). SMC124, EF77 and DB06-01 were found to be strong activators of human iNKT cells inducing a Th1-skewed cytokine response, thus of great therapeutic importance. The well-studied structural analogues of α-GalCer, OCH and C20:2, harbouring a shorter sphingosine chain and trimmed acyl chain, respectively, also activated iNKT cells significantly leading this time to Th2 cytokine polarisation (41–43). Later, the novel α-Galactosylsphingamides possessing an amide bond in their sphingosine chains were synthesised (44) and they exhibited poor iNKT cell agonism (**Figure 1**). The structural analysis of the ternary complex showed that the galactose moiety of the α-galactosylsphingamide was very slightly displaced within the CD1d binding groove. Overall, the modifications introduced in both aliphatic chains did not impact significantly the overall positioning of the galactose headgroup nor of the TCR CDR loops (**Figure 2F**).

## 5 6′′-OH galactose modified α-GalCer analogues

The NKT TCR-CD1d-lipid crystal structures clearly established that the interactions between the iNKT TCR and α-GalCer were exclusively mediated by a hydrogen bond network between the 2′′-, 3′′- and 4′′-OH of the galactose moiety and the iNKT TCRα chain. The 6′′-OH is the only galactose hydroxyl that is permissive for interaction with the TCR, and thus exploring the introduction of chemical modifications at this key position has been the focus of recent research initiatives for the development of novel potent anti-tumor glycolipids (**Figure 1**) (45, 46). These synthetic analogues shared the overall chemical features of α-GalCer except for the 6′′-OH that was replaced by a variety of bulkier aromatic chemical groups such as napthylurea (NU-α-GalCer) (**Figure 1**) (30). Here, the crystal structures of the mouse iNKT TCR in complex with mCD1d-NU-α-GalCer and mCD1d-BnNH-GSL-1’ revealed also that the TCR docked conservatively in a parallel fashion over the F′-pocket of the mCD1d-Ags (30) (**Figure 2B**). Relative to the α-GalCer, the overall position of the galactose moiety in NU-α-GalCer was highly conserved, thus maintaining all the key interactions with the TCR. Most interestingly, the napthylurea group did not make any contact with the TCR and was found to be bound within the A′-pocket of the mCD1d antigen binding groove in an ‘induced fit’ mechanism whereby the α1- and α2-helices of CD1d underwent structural rearrangements to create a hydrophobic cavity large enough to accommodate the bulky 6′′-substitution. However, this so-called ‘third anchor model’ of NU-α-GalCer in mCD1d was not observed in the BnNH-GSL-1’ ternary complex whereby the galactose moiety was slightly repositioned towards the CD1d molecule resulting in a loss of a hydrogen bond between the TCR and the 4′′-OH of the galactose. NU-α-GalCer exhibited also promising results *in vitro* and *in vivo* whereby the analogue could generate a strong Th1-biased immune response and was as effective as α-GalCer to suppress the progression of tumor metastases in a murine model (30). The fine-tuning of this encouraging immune response was further explored with the recent development and characterization of several synthetic molecules highly analogous to NU-α-GalCer (4ClPhC-α-GalCer, NC-α-GalCer, and PyrC-α-GalCer) (**Figure 1**) (32, 47). In this series of compounds, the C6′′ amide linker was replaced by a carbamate linker to confer more flexibility to the 6′′-OH modified moiety. Here, similarly to NU-α-GalCer, the stability of these analogues within CD1d was enhanced and this was correlated with an improved antitumoral potency in both humans and mice. Interestingly, whilst PyrC-α-GalCer exhibited superior antitumoral activity than NU-α-GalCer, the crystal structure of the iNKT TCR-mCD1d-PyrC-α-GalCer revealed that the pyridinyl carbamate moiety at the C6′′ position did not form a ‘third anchor’ with CD1 but instead mediated additional interactions with the iNKT TCR (**Figure 2B**). The recent development of glycolipid-peptide (GLP) conjugates wherein the C6′′-OH of α-GalCer has been attached to an immunogenic peptide antigen demonstrating co-stimulation of iNKT cells and peptide-specific CD8^+^ T cells offering protective immunity has been efficacious (48). Recently, the α-GalCer analogue AH10-7 that contained dual modifications including a C6′′-modified hydrocinnamoyl ester and the absence of a C4′′-OH group in the sphingosine base was synthesised in an effort to recover maximal Th1-biased stimulation of iNKT cells (49). Here, molecular studies revealed that the position of the galactose headgroup of AH10-7 was identical to that of KRN7000. However, additional molecular interactions were mediated by the hydrocinnamoyl ester moiety within the CD1d binding groove that may play an important role in offering an increased stability of the CD1d-AH10-7 complex for TCR recognition. The lack of a C4′′-OH group in the sphingosine base did not impact on the overall positioning of the sphingosine tail within the F′-pocket of CD1d as also observed within the recently reported crystal structures of CD1d in complex with *Bacteroides fragilis* produced α-GalCers (BfaGCs) (50). Overall, the chemical modifications introduced in the 6′′-OH galactose moiety did not drastically impact on the overall positioning of the galactose moiety (**Figure 2G**). Nevertheless, some of the modifications provided further molecular contacts with CD1d residues that may contribute to higher stability of the analogue within the CD1d binding groove.

## 6 Conclusions and future perspectives

Collectively, these insights into the molecular recognition of α-GalCer analogues by the iNKT TCR clearly revealed that the TCR typically maintained a universally conserved binding topology over the CD1d-lipid binding groove regardless of the α-GalCer analogue to be presented by CD1d. The CDR1α and CDR3α loops of the iNKT TCR and the galactose headgroup of the α-GalCer analogues generally adopted very similar positions within all the ternary complexes and subtle changes in the molecular interactions at the TCR/ligand interface were observed. Despite considerable efforts, the translation of immune responses from preclinical models to humans has been challenging largely due to the lower abundance of iNKT cells in humans and the sensitivity of iNKT cell anergy, wherein their frequent restimulation is not possible. To achieve the optimal therapeutic profile of these analogues, future research may need to progress in developing analogues with chemical modifications that may further enhance the overall stability and prolong half-life with desired outcomes and in establishing their role in humanised mouse models before moving to clinical trials in patients. Further, given the existence of other ‘atypical’ NKT cell subsets (γδ and δ-αβ T cells), it may also be important to further understand the impact of such modifications on the entire CD1d-α-GalCer reactive TCR repertoire beyond the type I NKT cells.
