# Corrigendum: State of play in the molecular presentation and recognition of anti-tumor lipid-based analogues

**DOI:** 10.3389/fimmu.2025.1574591

**Published:** 2025-02-27

**Authors:** T. Praveena, Jérôme Le Nours

**Affiliations:** Infection and Immunity Program and Department of Biochemistry and Molecular Biology, Biomedicine Discovery Institute, Monash University, Clayton, VIC, Australia

**Keywords:** CD1d, glycolipids, iNKT cells, α-GalCer, tumor, immunotherapy

In the published article, there was an error in **Figure 2** as published. In **Figure 2B**, the annotations for the last two ternary structures (last 2 panels) were inverted, namely, (iNKT TCR-mCD1d-PyrC-α-GalCer - PDB code: 4IRS) and (iNKT TCR-mCD1d-EF77 - PDB code: 4Y4K) were inadvertently swapped. The corrected **Figure 2** and its caption appear below.

**Figure 2:**
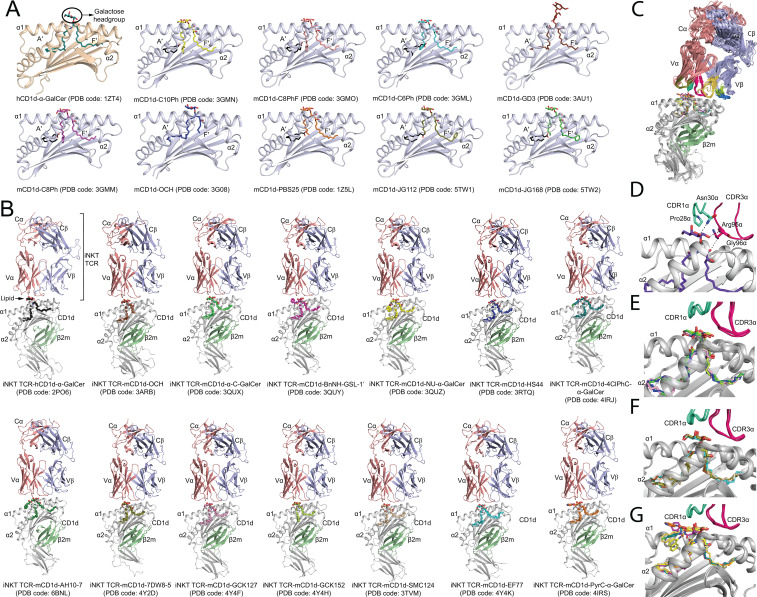
Molecular insights into the presentation and recognition of α-GalCer analogues. **(A)** Cartoon representation of the binding groove of CD1d presenting α-GalCer and modified α-GalCer. The PDB code for each binary complex crystal structure is indicated. The α1/α2 domain forming the hydrophobic binding groove (A′- and F′-pockets) of human CD1d (hCD1d) and mouse CD1d (mCD1d) are shown as cartoon representation in wheat and light blue, respectively. The bound α-GalCer and modified α-GalCer are shown as coloured sticks. Spacer lipids are shown as black sticks. **(B)** Cartoon representation of the crystal structure of iNKT TCR-CD1d-α-GalCer analogues ternary complexes deposited in the protein databank (PDB). CD1d, grey; TCRα, salmon; TCRβ, light blue; β2-microglobulin (β2m), green. The PDB code of the crystal structures are indicated. **(C)** Overall superposition of iNKT TCR-CD1d-α-GalCer analogues ternary crystal structures available in the PDB database. **(D)** Molecular interactions between the iNKT TCR and α-GalCer. Hydrogen bonds are shown as dashed lines. **(E)** Superposition of the bound O-glycosidic linkage modified α-GalCer. **(F)** Superposition of the bound acyl and phytosphingosine chains modified α-GalCer. **(G)** Superposition of the bound 6′′-OH galactose modified α-GalCer. The overall positioning of the CDR1α and CDR3α loops of the iNKT TCR are also shown in **(E–G)**.

The authors apologize for this error and state that this does not change the scientific conclusions of the article in any way. The original article has been updated.

